# Categorical Auditory Working Memory in Crows

**DOI:** 10.1016/j.isci.2020.101737

**Published:** 2020-10-27

**Authors:** Lysann Wagener, Andreas Nieder

**Affiliations:** 1 Animal Physiology Unit, Institute of Neurobiology, University of Tübingen, Auf der Morgenstelle 28, 72076 Tübingen, Germany

**Keywords:** Behavioral Neuroscience, Sensory Neuroscience, Cognitive Neuroscience

## Abstract

The ability to group sensory data into behaviorally meaningful classes and to maintain these perceptual categories active in working memory is key to intelligent behavior. Here, we show that carrion crows, highly vocal and cognitively advanced corvid songbirds, possess categorical auditory working memory. The crows were trained in a delayed match-to-category task that required them to flexibly match remembered sounds based on the upward or downward shift of the sounds' frequency modulation. After training, the crows instantaneously classified novel sounds into the correct auditory categories. The crows showed sharp category boundaries as a function of the relative frequency interval of the modulation. In addition, the crows generalized frequency-modulated sounds within a category and correctly classified novel sounds kept in working memory irrespective of other acoustic features of the sound. This suggests that crows can form and actively memorize auditory perceptual categories in the service of cognitive control of their goal-directed behaviors.

## Highlights

• Crows performed a delayed match-to-category task with frequency modulated sounds
• Crows classified novel sounds into upward or downward modulated sound categories
• Crows showed sharp category boundaries and within-category generalization
• Crows can actively memorize auditory perceptual categories for cognitive control

## Graphical Abstract

**Figure:**
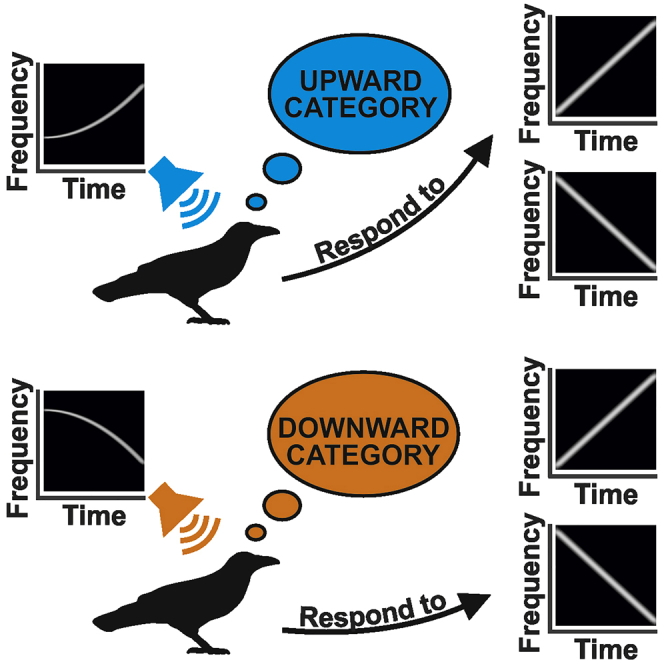

## Introduction

Categorical working memory, the ability to group sensory data into behaviorally meaningful classes and to maintain them active in working memory for a future goal, is key to intelligent behavior (Miller et al., 2018). It allows humans and animals to classify, memorize, and process sensory information efficiently. This enables humans and cognitively advanced animals to quickly adapt to new situations (Miller et al., 2003).

So far, categorical working memory in animals has primarily been demonstrated in the visual domain. In classical working memory tasks, monkeys and crows flexibly switch between remembered visual categories, such as “leftward versus rightward motion” (Zhou and Freedman, 2019), “cats versus dogs” (Freedman et al., 2001), or “same versus different” (Wallis et al., 2001; Veit and Nieder, 2013). However, whether categorical working memory is also found in the auditory domain is currently unknown.

This lack of knowledge about auditory categorical working memory is surprising because this cognitive capability is essential during goal-directed audio-vocal communication. In a telephone group call, for instance, we categorize speech signals as belonging to a specific individual and maintain this auditory category in working memory in order to match it to subsequent speech signals of the same speaker while following a conversation. Undoubtedly, also animals that rely on elaborate audio-vocal communication would benefit from this cognitive ability. Unfortunately, most animals are notoriously difficult to train on complex auditory tasks (Plakke and Romanski, 2016). Currently it is therefore rarely studied whether animals can actively maintain auditory categories in working memory (Tsunada et al., 2011).

As true vocal learners, songbirds face many challenges of acoustic communication with speaking humans (Mooney, 2009). To follow an audio-vocal communication, songbirds need to recognize communication partner's characteristics, such as sex, group membership, or identity (Wascher et al., 2015; Brecht and Nieder, 2020). In short, songbirds rely both on acute hearing and cognitive abilities to classify a multitude of raw acoustic stimuli and memorize this information across time (Nieder and Mooney, 2020). Indeed, songbirds are known to perceive sounds in a categorical way (Dooling et al., 1995; Burgering et al., 2019). In addition, they show working memory for auditory items comparable with humans (Zokoll et al., 2007; Comins and Gentner, 2010). However, whether birds can combine both capabilities to actively memorize auditory categories for future goal-directed behavior is unknown, and this capability is barely studied in animals in general. Here, we addressed this issue in carrion crows, a vocal corvid songbird that can be trained on complex tasks (Nieder, 2017; Brecht et al., 2019; Nieder et al., 2020) requiring conceptual understanding and behavioral flexibility (Veit et al., 2015; Moll and Nieder, 2014; Smirnova et al., 2015; Ditz and Nieder, 2016a).

## Results

**Figure 1:**
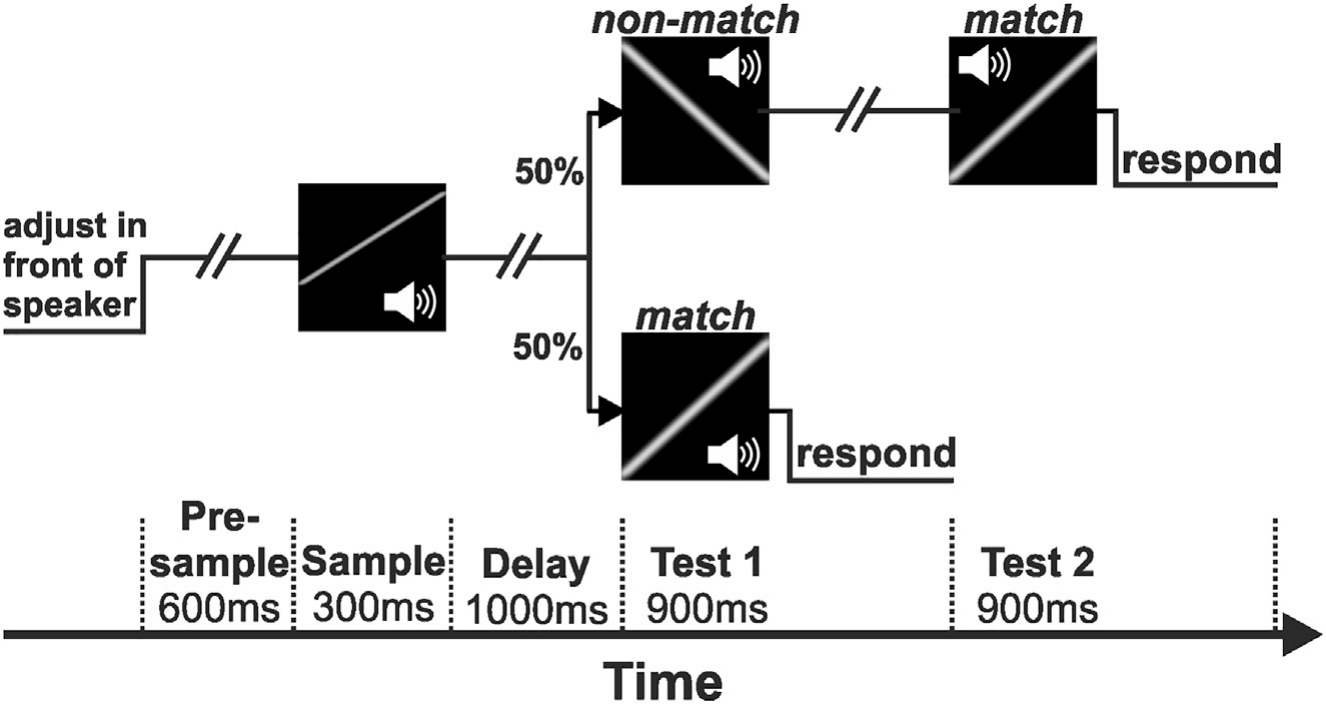
Task Design The trial began when the crow adjusted its head in front of the speaker and screen (by entering an infra-red light barrier) in response to a central visual Go-cue displayed on the screen. After the crow had adjusted its head, the screen turned blank for the rest of the trial. A silent pre-sample period (600 ms) was followed by a frequency-modulated sample sound that was played for 300 ms. The sample was followed by a 1s silent delay and then by a choice (Test) sound (900 ms). *Lower trial end-sequence:* If the category (upward or downward FM) of Test1 matched that of the sample (“match” condition), the crow had to move its head and leave the infra-red light barrier to the Test1 sound within the 900 ms response time (shifted by 100 ms relative to Test-onset) to obtain a food reward. *Upper trial end-sequence:* If Test1 was a nonmatch (“non-match” condition), a match followed as Test2, which required a head movement for a reward. There were an equal number of match and nonmatch trials and they were randomly interleaved.

We trained crows on a delayed match-to-category task with sounds (Figure 1). In this task, the crows indicated whether a test sound was a categorical match to a previously presented and memorized sample sound. In each trial, the crows evaluated and maintained the direction of frequency modulation (FM) of the sample sounds in working memory to subsequently match them to the upward or downward modulated sound categories. Since individual trials presented varying sound combinations, the crows had to flexibly categorize what they heard on a trial-by-trial basis.

**Figure 2:**
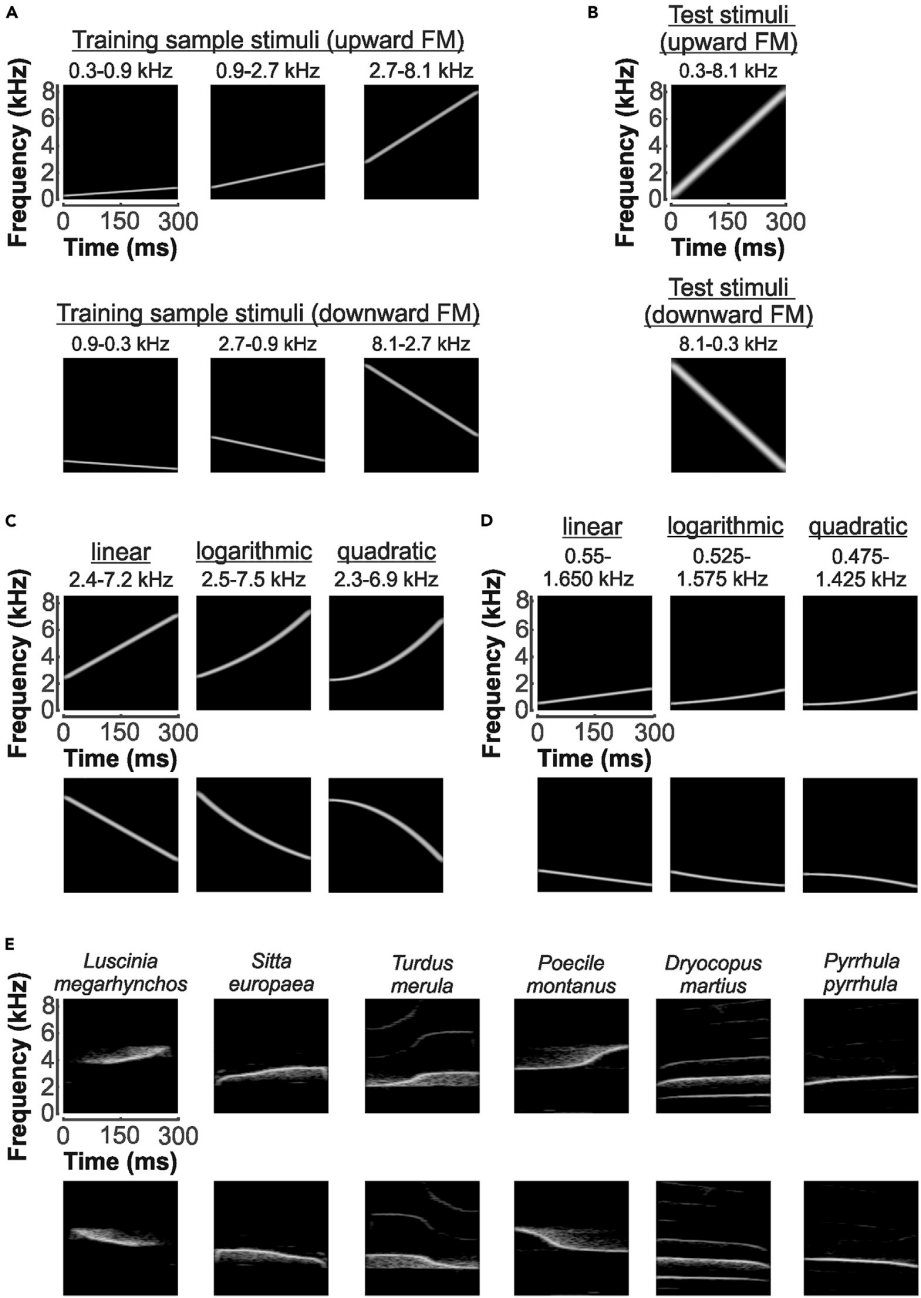
Auditory Stimuli Depicted as Sonagrams (A) The familiar sample stimuli for training the crows were three upward and three downward linear FM sweeps. (B) The same two upward and downward FM sweeps were used as test stimuli. (C) Examples of new probe sample stimuli with frequency interval ratios of 3:1 (1.6 octaves). Linear, logarithmic, and quadratic sweeps in a high-frequency range are shown. Top row displays upward FM sweeps, bottom row shows the corresponding downward FM sweeps. (D) Same layout as in (C), showing linear, logarithmic, and quadratic sample probe stimuli in a low-frequency range. (E) Probe sample stimuli consisting of segments of bird vocalizations. Six representative examples of the 20 stimuli are depicted. Top row shows upward, bottom row the corresponding mirrored downward stimuli.

The crows were first trained to match six fixed FM sample stimuli (“training stimuli,” three upward and three downward sweeps) to the upward or downward categories (Figures 2A and 2B). The frequency range of the upward and downward FM stimuli together covered the entire hearing range of crows (Jensen and Klokker, 2006). Once the crows reached reliable performance with these training sample stimuli, novel probe sample stimuli were occasionally inserted in the daily sessions (Figures 2C–2E), while the crows continued to discriminate the training stimuli as background task. Both crows performed 10 successive sessions with randomly interleaved training and probe stimuli.

**Figure 3:**
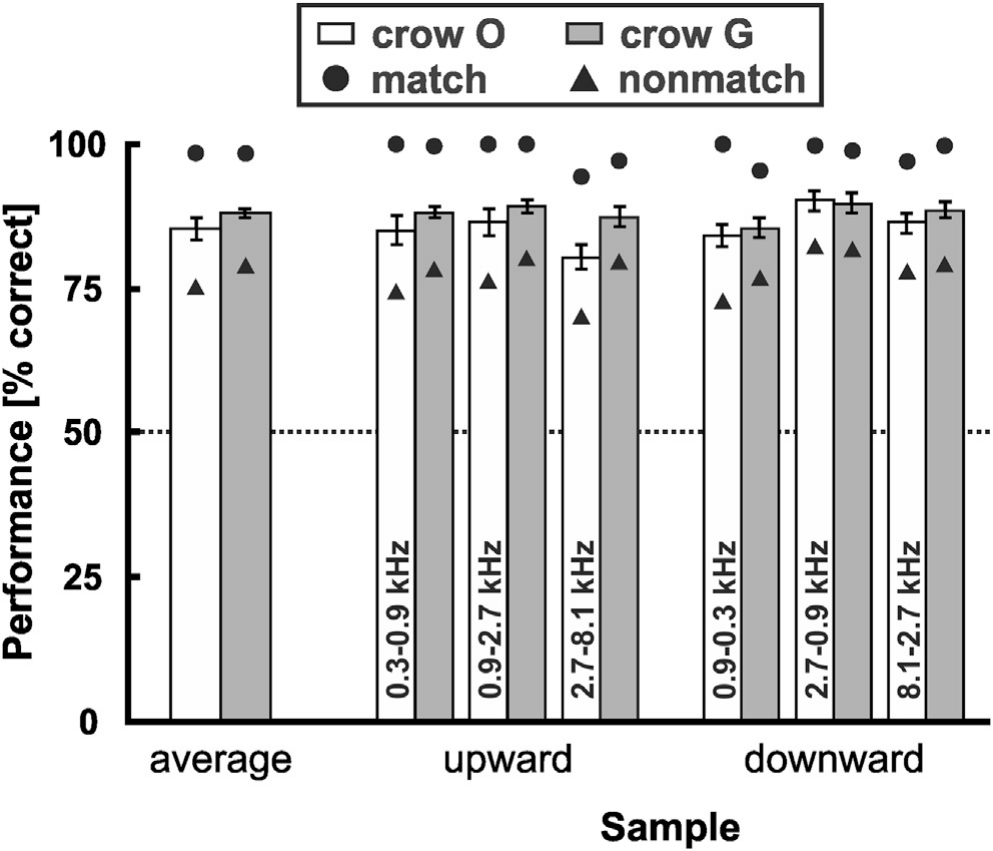
Performance to Familiar Training Stimuli Both crows responded significantly above chance (dashed horizontal line at 50% performance) to upward and downward FM samples. Columns represent mean performance values averaged across match and non-match trials (error bars: standard error of the mean), circle and triangle symbols reflect mean performance for match and non-match trials separately.

For the training sample stimuli, crow O performed an average of 430 correct background trials per session (±52 STD, n = 10) and reached mean performance of 85.2% (±6.1% STD across sessions) (Figure 3). Crow G on average accomplished 426 correct background trials per session (±36 STD, n = 10), with a mean performance of 87.7% (±2.5% STD) (Figure 3). The average performance of both crows with the background stimuli in each daily session was significantly above the 50%-chance level (each binomial test, p < 0.001). Owing to the temporal succession of the matching test stimulus in the “match” versus “non-match conditions,” both crows had a bias toward responding to test1, resulting in systematically higher performances during match trials (see separate data points for match and non-match performances in Figure 3). However, not only match but also all non-match performances separately were significantly above chance for both crows and all conditions (each binomial test, p < 0.001). The crows' mean performances for each of the six training sample stimuli was indifferent (each one-way ANOVA, p > 0.05).

**Figure 4:**
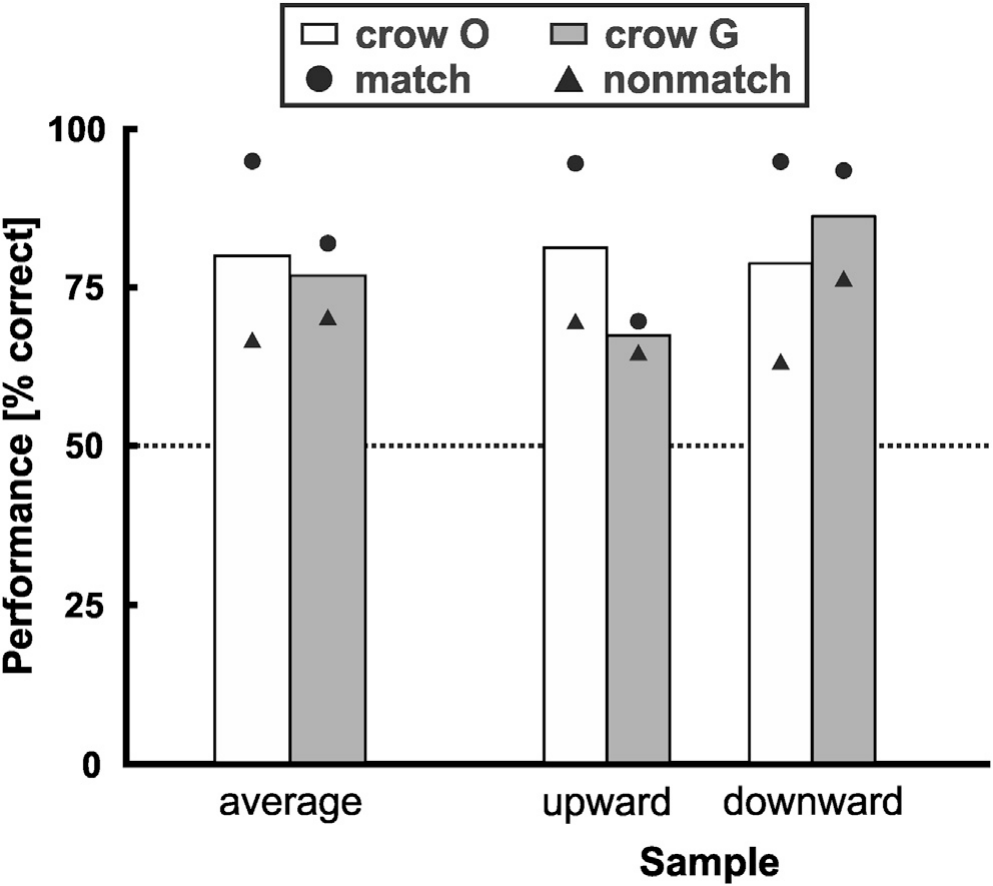
Overall Performance to Novel Probe Stimuli Both crows responded significantly above chance to upward and downward FM probe samples. Columns represent mean performance values averaged across match and non-match trials (error bars: standard error of the mean), circle and triangle symbols reflect mean performance for match and non-match trials separately.

Next, we tested whether the crows could generalize novel FM sounds they had never heard before to the appropriate categories and thereby would demonstrate a conceptual grasp of sound categories. To that aim, we occasionally introduced novel probe sample sounds (12% of the trials) in the daily sessions with the training sounds (the remaining 88% of the trials). Four classes of novel probe sample sounds were presented: three classes of pure-tone FM sweeps with linear (where frequency changes linearly with time), logarithmic (where frequency changes logarithmically with time), and quadratic (where frequency changes quadratically with time) frequency trajectories, and frequency-modulated segments of bird vocalizations. The frequency interval ratios of the pure-tone probe sweeps were 2:1 (1 octave), 3:1 (1.6 octaves) (examples in Figures 2C and 2D), and 4:1 (2 octaves). The mean frequency interval ratio of probe bird vocalizations was 1.47:1 (around half an octave), on average (Figure 2E). The number of upward and downward-modulated probe stimuli was balanced. Because the goal was to test whether the crows could instantaneously transfer the FM categories without additional learning, we only analyzed responses to the first presentation of each unique probe stimulus.

Across all probe stimuli and classes, both crows showed a significant category transfer (each binomial test, p < 0.001, *n* = 160) (Figure 4). For all ten sessions together, crow O responded 80% (128/160 trials) and crow G responded 77% (123/160) correctly across all probe stimuli (which was comparable with the performance with training stimuli in crow O but significantly worse in crow G; binomial test, p < 0.05). To ensure that the transfer was made for each of the two categories, we analyzed the performance to upward and downward FM probes separately. Again, both crows performed well above chance level for both categories separately (each binomial test, p < 0.01, *n* = 80) (Figure 4). Crow O responded correctly in 81% and 79% of the trials presenting upward and downward FM probe stimuli, respectively. Crow G responded correctly in 68% and 86% of the trials presenting upward and downward FM probe stimuli, respectively. Again, not only match but also non-match performances separately were significantly above chance for both crows and all conditions (each binomial test, p < 0.05), except for one (downward for crow O, binomial test, p = 0.059).

**Figure 5:**
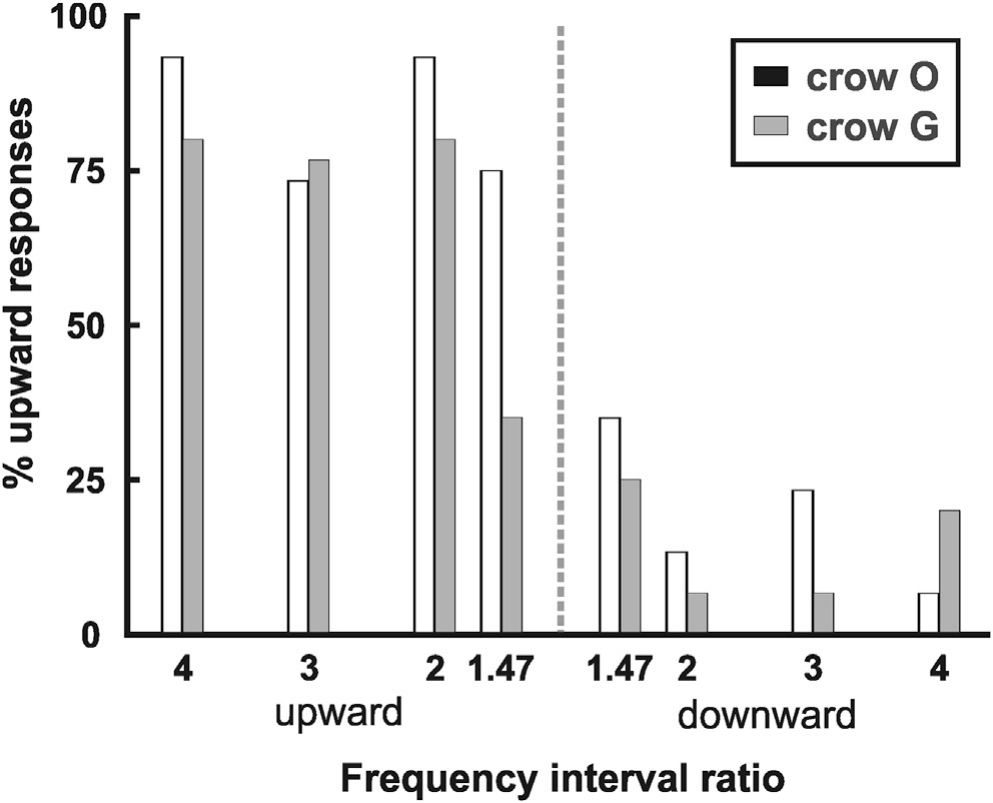
Performance Relative to Category Boundary Categorization performance to probe stimuli of different frequency interval ratios of upward and downward FM sounds. Performance is depicted as percent correct classification as “upward” category. Vertical dashed line indicates the category boundary at a frequency interval ratio of 1.

Categorization is characterized by sharp category boundaries and within-category generalization. We first analyzed performance as a function of distance to the category boundary. The physical dimension for categorization of FM sounds into the perceptual “upward” and “downward” categories is the frequency interval ratio of the sounds. A frequency interval ratio of 1 (i.e., no change in frequency with time) demarcates the category boundary relative to which upward versus downward frequency-modulated sounds of increasing frequency interval ratio can be classified into the FM categories upward versus downward. Figure 5 depicts the crows' judgments of upward category as a function of the probes' frequency interval ratios. As expected for categorical behavior, the crows classified rising FM sounds into the upward category and falling FM sounds into the downward category, with an abrupt switch of performance at the category boundary. Performance for probe sweeps at high-frequency interval ratios (4:1, 3:1, and 2:1) (each binomial test, p < 0.001, *n* = 30 for ratios of 4:1 and 2:1, respectively, *n* = 60 for a ratio of 3:1). The performance of crow O was 93%, 75%, and 90% for ratios of 4, 3, and 2, respectively. The performance of crow G was 80%, 85%, and 87% for ratios of 4, 3, and 2, respectively. As expected, categorization with probe bird vocalizations that had the lowest frequency interval ratio of all probe sounds near the category boundary became increasingly more difficult for the crows. Crow O correctly categorized the probe bird vocalization sounds (70%; binomial test, p < 0.01, n = 40), whereas crow G showed a tendency but did not reach significance (55%; binomial test, p = 0.32, n = 40). Overall, however, the crows categorized novel sounds correctly into the appropriate categories, with categorization performance suffering close to the category boundary.

**Figure 6:**
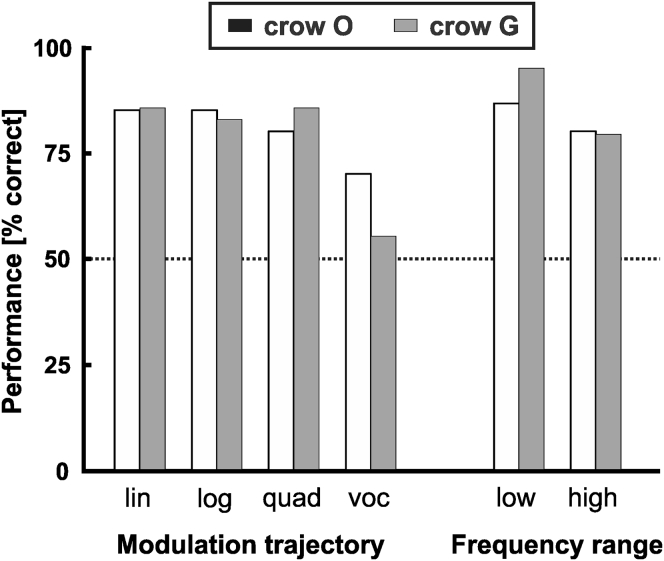
Performance to Probe Stimuli as a Function of Modulation Trajectory and Frequency Range Chance level is again 50% performance.

Next, we investigated within-category generalization performance. Within-category generalization predicts that performance is independent from the acoustic details of the FM sound, such as the modulation trajectory and the frequency composition of the sounds. To that aim, we separately analyzed and compared performance to the four probe stimulus classes (linear, logarithmic, quadratic pure-tone FM sweeps, and bird vocalization segments). Both crows showed high performance to all probes containing FM sweeps of different trajectories. (linear: crow O 85%, crow G 85%; logarithmic: crow O 85%, crow G 83%; quadratic: crow O 80%, crow G 85%) (each binomial test, p < 0.001, n = 40) (Figure 6). As mentioned above, the bird vocalization probes that exhibited only mild frequency modulation were close to the category boundary and thus more difficult for the crows. To summarize, for probe sounds with distinct frequency modulation, the crows categorized performance was independent from the type of modulation trajectory.

In addition, we investigated whether the frequency range of the 120 pure-tone probe stimuli (linear, logarithmic, and quadratic sweeps) had an influence on behavior. Half of these stimuli had a frequency between 0.3 and 2.7 kHz and were therefore assigned to the group of “low-frequency” stimuli. The other half had a frequency between 0.9 and 8.1 kHz and were grouped as “high-frequency” stimuli. Stimuli including frequencies in the overlapping range of 0.9–2.7 kHz never contained both frequencies lower than 0.9 kHz and higher than 2.7 kHz. The crows performed well above chance regardless of the frequency range of the sample stimuli (each binomial test, p < 0.001, *n* = 60) (Figure 6). Crow O responded correctly in 87% and 80% of low frequency and high frequency trials, respectively. Crow G responded correctly in 92% and 77% of low frequency and high frequency trials, respectively. Thus, the crows showed robust within-category generalization irrespective of the frequency range of the probe sounds.

## Discussion

Our data show that crows possess categorical auditory working memory. They are able to maintain the FM categories upward and downward in working memory to master an auditory delayed match-to-category task. As a sign of categorical generalization and transfer, the crows instantaneously and without further training matched the remembered novel sample sounds correctly to the upward and downward FM categories, irrespective of other sound parameters. The crows' behavior showed the diagnostic characteristics of categories, namely, sharp category boundaries and within-category generalization: the crows categorically classified the continuous direction of FM into upward and downward while ignoring other sound parameters (such as spectral composition, frequency intervals, or modulation trajectory of the novel sample sounds) within one FM sound category. This suggests that the crows only memorized the direction of the FM, not the other varying sound parameters, when categorizing sounds from working memory.

### Auditory Categorization in Birds

Birds have also been shown to discriminate and classify complex sounds. Vocal learners, in particular, rely on acute audition and are known to perceive sounds in a categorical way (Dooling et al., 1995; Burgering et al., 2019). Even pigeons, non-songbirds with an unlearned vocal repertoire, are able to make same/different discriminations across a wide variety of auditory stimuli (Murphy and Cook, 2008; Cook and Brooks, 2009; Cook et al., 2016) and can learn to discriminate among music-derived acoustic elements and sequences (Brooks and Cook, 2010; Hagmann and Cook, 2010; Brooks and Cook, 2010; Cook, 2017). However, previous experiments did not require the birds to flexibly switch between auditory categories or remember auditory categories in working memory. In these studies, the birds were typically tested in Go/NoGo or forced choice tasks without a delay period. Both temporal and spectral changes in the sounds could be exploited.

Birds are known to categorize complex sounds, such as human speech sounds, based on temporal differences. For instance, budgerigars place vowels /i/, /a/, /e/, and /u/ in phonetically appropriate categories in spite of variation in who is talking and their gender (Dooling and Brown, 1990). When working with synthetic phoneme continua of speech sounds, budgerigars exhibit perceptual phonemic boundaries near the human boundaries for /ba/-/pa/, /da/-/ta/, /ga/-/ka/, /ra/-/la/, and /ba/-/wa/ (Dooling et al., 1995; Dent et al., 1997). Similar perception of speech sound categories has also been shown in quails and zebra finches (Burgering et al., 2019; Kleunder et al., 1987; Ohms et al., 2010). Because the phoneme boundaries rely on temporal differences (or “voice onset time” between the vowel and the consonant), these data suggest that not only sound frequency but also sound timing plays an important role in birds' capability to categorize sounds.

Besides temporal factors, also the spectral composition of sounds can be exploited by birds. In a series of experiments, several songbird species (primarily European starlings) have been shown to perceive pitch relations in a simple tonal melody (Hulse and Cynx, 1985). In particular, songbirds can classify rising as opposed to falling pitch patterns. However, these songbirds preferentially discriminated tonal patterns according to the absolute frequency of the individual element tones in the patterns; they failed to transfer discrimination to a novel frequency range when the training frequency range was shifted. Only when the experimental conditions severely constrained the use of pattern element cues did the songbirds use pitch relations as a secondary strategy (Hulse and Cynx, 1986; Hulse et al., 1984; Braaten et al., 1990). Data like these lead to the conclusion that birds, unlike humans, cannot generalize relative pitch discrimination to new frequencies, thus lacking a conceptual grasp of frequency modulation in complex sounds. However, our data suggest that corvid songbirds can indeed form a conceptual understanding of upward and downward frequency modulation, irrespective of frequency composition.

### Auditory Working Memory in Birds

Auditory working memory capabilities have only rarely been studied in birds, mainly because it is difficult to train birds—and nonhuman animals in general—to perform auditory working memory tasks that are similar to those used in the study of visual memory (Plakke and Romanski, 2016). Nonetheless, a few studies show that European starlings exhibit auditory working memory and show interesting similarities and differences when compared with humans (Zokoll et al., 2007, 2008a, 2008b; Comins and Gentner, 2010). For example, the classical finding of a decay of working memory with increasing delay times in humans and other animals could be reproduced in starlings (Zokoll et al., 2008a, 2008b). In contrast to humans, however, starlings benefited from repeated presentations of sample sounds. Our study adds to these insights by showing that songbirds maintain not only specific sounds in working memory but also overarching auditory categories. Overall, songbirds are therefore valuable models for investigating not only mechanisms of auditory signal processing but also cognitive control functions in the auditory domain.

### Categorization of Bird Vocalizations

In contrast to novel pure-tone FM sweeps, novel segments of frequency-modulated bird vocalizations were more difficult to categorize for the crows. One crow reached significant categorization (albeit with less precision than with the pure-tone probes), whereas the other crow showed a tendency but failed significance. Most likely, this difficulty was due to the vocalization segments having the lowest frequency interval ratio of all probe sounds, a ratio that was closest to the category boundary. In addition, the vocalizations were acoustically more complex and richer. Some of them contained broadband noise that potentially could have masked the FMs and additional harmonics that might have distracted the crows. Overall, however, these data suggest that corvids can categorize and remember animal sounds in order to adapt their behavior.

The capability to memorize sound categories may also have adaptive advantages in a world in which objects and events are characterized by multi-modal signals. The semantic grouping of a multitude of unique stimuli into uni-modal categories facilitates the association with stimuli from other sensory modalities that characterize the same members of a class. For instance, social songbirds need to group conspecifics into different categories based on sex, relatedness, or group membership in order to adjust their behavioral responses. Crows recognize group members by identity congruence between visual presentation of a group member and the subsequent playback of a contact call (Kondo et al., 2012). Because corvids can recognize individuals by sound (Wascher et al., 2015) or sight alone (Kondo et al., 2010), the most parsimonious explanation is that they first categorize acoustic and visual stimuli as belonging to an individual and later associate the auditory and visual categories for cross-modal audiovisual recognition of group members. The brain of crows is able to associate stimuli across modality and time (Moll and Nieder, 2015, 2017). However, whether this extends also to more cognitive cross-modal categories remains to be explored.

### Categorization of Pure Auditory Frequency Modulation in Mammals

The categorical discrimination of sounds based on pure frequency modulation has been demonstrated convincingly in a mammal, the Mongolian gerbil (Wetzel et al., 1998; Ohl et al., 2001). In this positive-reinforcement Go/NoGo task, the effects of conditioned fear (CS+) based on FM categories were tested. The gerbils had to change compartments in a shuttle box during ascending FMs (CS+) presentation to avoid foot shock. The gerbils were able to discriminate FM tones by modulation direction and, after familiarization with a number of different FM pairs, transferred the ascending-descending concept to stimuli not heard before (Wetzel et al., 1998). A similar conditioning approach was used in categorization studies with ferrets (Yin et al, 2016, 2020); in one study, individual ferrets were trained to discriminate downward sequences (the target sequence) from upward sequences (the reference sequence), or vice versa (Yin et al., 2010). In both approaches, gerbils and ferrets thus discriminated a fixed FM category stored in long-term memory from deviating sounds.

Although these experiments clearly show perceptual categorization of FMs in gerbils and ferrets, they required the animals neither to flexibly switch between different auditory categories nor to maintain the switching categories in auditory working memory. To address both cognitive aspects, we therefore trained crows on a delayed match-to-category task. This task not only tested the formation of one FM category against other sounds but probed the conceptual flexibility of the crows to switch between rewarded and unrewarded FM categories on a trial-by-trial basis. In addition, the crows could not have succeeded without a working memory for the auditory categories.

### Categorical Auditory Working Memory in Monkeys

Categorical auditory perception and working memory have been reported in macaque monkeys. Using a delayed match-to-sample protocol, monkeys were trained to report by an eye movement whether two consecutive human-speech sounds (“dad” versus “bad”) or a series of morphed versions of these sounds belonged to the same or different category (Tsunada et al., 2011). The behavioral data showed that monkeys perceived these morphed speech sounds categorically; despite the gradual variation of the acoustic stimulus, the monkeys reliably assigned the morphs to one of the two categories and exhibited a sharp transition boundary between morphed sounds being perceived as dad rather than bad.

Whether the monkeys could also categorize novel morph sounds or other types of speech sounds as a sign of abstract categorization was not tested in this study. We tested this in the current study and found that the crows instantaneously categorized the remembered novel sample sounds correctly to the upward and downward FM categories, irrespective of other sound parameters. Crows can transfer the semantic grouping criteria they learned to novel and acoustically distinct sounds.

It is worth mentioning that the auditory working memory capacity of monkeys seems to be surprisingly limited and prone to interference. When rhesus monkeys were tested in an auditory delayed match-to-sample task equivalent to the task structure of the current study in which either the first (match condition) or the second test stimulus (nonmatch condition) could be a match and required a response, marked performance differences between the two conditions surfaced. Performance was accurate whenever a match followed the sample directly, but it fell precipitously if (one or two) nonmatch stimuli intervened between sample and match. This drop in accuracy was found to result from an “overwriting” effect, i.e., a retroactive interference from the intervening nonmatch stimulus that was far greater than that observed previously in delayed match-to-sample tasks with visual stimuli. The authors concluded that the monkeys' performance depended on the retention of stimulus traces in the passive form of short-term memory rather than on active working memory (Scott et al., 2012, 2013).

Our data from crows only allow an evaluation of this issue for zero (match condition) or one interfering stimulus (nonmatch condition). The data plotted in Figures 3 and 4 show a similar tendency, namely, a decline in accuracy in the nonmatch condition. Notably, crow G showed only a mild decline in the nonmatch condition when tested with novel probe stimuli (Figure 4). It is also worth mentioning that part (or all) of this performance decline may be due to the crows' bias to respond rather quicker (match condition) to receive a reward earlier. In addition, the performance and response pattern of crows for match and nonmatch conditions is comparable with those we see for visual categorization in delayed match-to-sample tasks (Ditz and Nieder, 2016a, 2016b, 2020; Wagener et al., 2018). Overall, the data suggest that the crows possess active working memory capacities also for auditory stimuli.

### Limitations of the Study

This study explored the crows' category generalization capabilities to a limited set of probe stimuli and found that the crows had more difficulty categorizing FM segments of bird vocalizations. One explanation for this finding is that vocalizations showed the smallest frequency interval ratio of all probe stimuli. However, compared with the pure tone training FM sweeps, vocalizations also showed additional harmonics. To demonstrate that crows can generalize FM categories to acoustically richer sounds, the application of multi-harmonic FM sweeps as training and probe stimuli would be helpful. In addition, and to further differentiate active working memory from potential passive short-term memory, the crows' performance when confronted with more than one distractor and for longer delays would be informative. Resistance against distraction over longer delay periods would corroborate the notion of auditory working memory in crows as it is regularly seen in the visual domain.

### Resource Availability

#### Lead Contact

Andreas Nieder, andreas.nieder@uni-tuebingen.de.

#### Materials Availability

This study did not generate new unique Materials.

#### Data and Code Availability

Original data have been deposited to Mendeley: https://doi.org/10.17632/38x8ktrx7y.1.

## Methods

All methods can be found in the accompanying Transparent Methods supplemental file.

## Supplemental Information

Document S1. Transparent Methods
