# Supplementary material for: Categorical Auditory Working Memory in Crows
Source: iScience. 2020 Oct 27;23(11):101737. doi: 10.1016/j.isci.2020.101737 (PMC7662871; doi:10.1016/j.isci.2020.101737)
Supplement: Document S1. Transparent Methods [file mmc1.pdf]

iScience, Volume *23*

Supplemental Information

Categorical Auditory Working Memory in Crows

Lysann Wagener and Andreas Nieder

Supplemental Information

Categorical auditory working memory in crows

Lysann Wagener and Andreas Nieder

TRANSPARENT METHODS

Subjects

Two 3 years old male carrion crows (*Corvus corone*) were used in this study. The crows were housed in social groups in indoor aviaries. During the training and testing period, the crows were on a controlled feeding protocol. Body weight was measured daily. Food was given as reward during the sessions. Water was *ad libitum* available in the aviary and during the experiments. All procedures were carried out according to the guidelines for animal experimentation and approved by the responsible national authorities, the Regierungspräsidium Tübingen, Germany.

Experimental setup

The birds were placed on a perch in front of a touchscreen monitor (3M Microtouch, 15“, 60 Hz refresh rate) in a darkened operant conditioning chamber (length 1 m, width 0.76 m, height 1 m). One speaker (VISATON B 200 – 6 Ohm) was used to play back the auditory stimuli. The speaker was located 0.6 m in front of the bird and behind the computer monitor. The behavior was controlled by the CORTEX system (National Institute of Mental Health, Maryland, USA) which also stored the behavioral data. An automated feeder delivered either mealworms (*Tenebrio molitor* larvae) or bird seed pellets upon correctly completed trials. An infrared light barrier was installed above the birds' head to which a reflector foil was attached. The crow had to keep its head still within the beam of the light barrier and thereby in front of the touchscreen throughout a trial.

Behavioral task

The crows were trained on a delayed match-to-category task in which they discriminated the direction of upward and downward frequency modulated (FM) sounds (**Fig. 1**). A crow started a trial by positioning its head in front of the monitor whenever a go-stimulus (small white cross) was shown on the screen. Head position was monitored by an infra-red light barrier, and the crows had to maintain the head still throughout the trial. Premature head movements terminated the trial and it was discarded. When the head was in the correct position in front of the monitor, the crows received auditory feedback and the go-stimulus on the screen turned into a white circle for 60 ms. For the further course of the trial the monitor remained black. After a 600 ms silent pre-sample phase, the auditory FM-modulated sample stimulus (300 ms duration) was played. This was followed by a 1000 ms silent delay period during which the crow had to memorize the direction of the frequency modulation (upward or downward) of the sample. In the following test phase, the crow had to match the direction of the FM in the sample to the test stimulus with the same FM direction (i.e. upward to upward FM, and downward to downward FM). If the direction of the FM matched, the crow had to respond by quickly moving its head out of the light barrier to receive a reward.

In 50% of the trials, the first test stimulus (test1) was the matching stimulus ('match condition'). In the other 50% of the trials, the test1-stimlus was a 'non-match' with a FM in the opposite direction of the sample's FM direction ('nonmatch condition'). In this case, the bird had to refrain from responding and wait with a response until the second test stimulus was played which was always a match. Both the test1- and the test2-periods were 900 ms in duration, with the 300 ms test1- and test2-stimuli played right at the beginning of the test-periods (so that the remaining 600 ms of the test-periods were silent). The response interval was shifted by 100 ms due to the inevitably reaction latency relative to physical stimulus onset. Responses to the 'nonmatch stimulus' and no response to either of the two test stimuli were considered as error and also not rewarded. Match and non-match conditions were balanced and pseudo-randomly presented. The crows were first trained with well-known training stimuli. Once the crows reached high performance, we tested if they were able to transfer the upward and downward FM categories to novel stimuli that were occasionally presented among the ongoing discrimination of the training sample stimuli.

Stimuli

A total of 168 auditory frequency modulated stimuli were used in this study. All stimuli had a duration of 300 ms and a 10 ms linear amplitude ramp at the beginning and the end.

Training stimuli. The crows were trained with a fixed set of 6 FM sample stimuli (3 upward and 3 downward sweeps). These ***training sample stimuli*** consisted of linearly rising or falling FM pure tones (**Fig. 2A**). The frequency range of the three upward training sample stimuli were 0.3-0.9 kHz, 0.9-2.7 kHz and 2.7-8.1 kHz. The identical frequency range of the three downward training sample stimuli was 0.9-0.3 kHz, 2.7-0.9 kHz and 8.1-2.7 kHz. Thus, each training sample stimulus had a bandwidth of 1.6 octaves. Each of these sample stimuli had to be matched to its corresponding matching test stimulus. A linearly FM-modulated sweep from 0.3-8.1 kHz was the match for upward FM stimuli, whereas a linear downward sweep from 8.1-0.3 kHz served as a match for downward FM stimuli (**Fig. 2B**).

Probe sample stimuli. Once the crows reliably discriminated and categorized the training stimuli, we tested their ability to transfer the upward and downward FM categories to novel sample sounds (probe stimuli). We tested a total of 80 probe stimulus pairs (each with upward and downward FM modulation) which the crows had never encountered before. Only responses to the first presentation of each unique probe stimulus – before the crows could learn a 'correct' response to these new stimuli - were analyzed. The test-stimuli remained the same as in the training trials.

The probe stimuli were grouped into four classes of FM sweeps: linear, logarithmic and quadratic FM modulation of pure tones, and FM-modulated bird vocalizations. Each of the four classes consisted of 40 unique stimuli (20 upward and 20 downward sweeps). All pure-tone sweeps (including the training, test and probe stimuli) were generated using a custom written MATLAB code. The sounds were saved as wav-files at a sampling frequency of 44.1 kHz.

$$\text{Linear: } f_i(300ms) = f_0 + \beta t \text{, where } \beta = (f_1 - f_0)/t_1$$

$$\text{Logarithmic: } f_i(300ms) = f_0 * \beta^t \text{, where } \beta = \left(\frac{f_1}{f_0}\right)^{\frac{1}{t_1}}$$

$$\text{Quadratic: } f_i(300ms) = f_0 + \beta t^2 \text{, where } \beta = (f_1 - f_0)/{t_1}^2$$

The pure-tone probe stimuli differed in frequency-modulation range and frequency content. The frequency-modulation ranges was quantified by the frequency interval ratio, which is the maximum frequency contained in the FM sound divided by the minimum frequency (f_{max} : f_{min}). The probe FM sweeps had frequency interval rations of 2:1 (1 octave), 3:1 (1.6 octaves; **Fig. 2C**) and 4:1 (2 octaves).

The frequency content was roughly divided into 'low' and 'high' frequencies. The 'low frequency' probe stimuli covered frequencies between 0.3-2.7 kHz (examples shown in **Fig. 2D**), whereas the 'high frequency' stimuli covered 0.9-8.1 kHz. Stimuli including frequencies in the overlapping range of 0.9 to 2.7 kHz were never both, lower than 0.9 kHz and higher than 2.7 kHz at once. Likewise, none of the stimuli laid exclusively within the overlap, so that each stimulus could be related to 'low' or 'high' based on whether it reached into the range of 0.3-0.9 kHz or 2.7-8.1 kHz, respectively.

The bird vocalization probe stimuli were excerpts of bird vocalizations (for example, *Parus major*, *Sturnus vulgaris*, *Buteo buteo*, *Alcedo atthis*) (downloaded from http://www.xeno-canto.org/) which have been recorded at 16-bit resolution and almost all a sampling rate of 44.1 kHz (except for two at 48 kHz and one at 16 kHz). These were further modified using Adobe Audition 3.0 and Audacity 1.0.0. From all vocalizations, a 300 ms segment covering a monotonic frequency change was extracted. The amplitude of the signal was equalized to the pure-tone stimuli and 10 ms ramps were added. Each vocalization probe stimulus was used

with its original FM-sweep direction (8/20 upward, 12/20 downward) for one FM category, and as a temporally inverted version for the other FM category. The average frequency interval ratio of the vocalization probe stimuli was 1.47:1 (± 0.25 STD).

Transfer to novel FM stimuli was tested during 10 sessions. In each session we used four different stimuli per probe class (linear, logarithmic, quadratic and bird vocalization sweeps) with two upward and two downward sweeps per class (or two probe stimulus pairs per probe class). The upward and downward sweep of each probe pair covered exactly the same frequency range. The pure-tone probe stimuli for each daily session were selected so that each session contained 2 'low frequency' and 2 'high frequency' linear, logarithmic and quadratic sweeps. For the first 5 sessions of the experiment, only pure-tone probe stimuli with a bandwidth of 1.6 octaves were used, whereas for the second 5 sessions stimuli with 1 and 2 octaves were used (6 of each in each session).

Each session consisted of an average of 577 completed pseudo-randomized trials for crow O and 566 completed trials for crow G. Of those, the familiar training sample stimuli were presented in 88% of the trials and probe sample stimuli were presented pseudo-randomly in the other 12% of the trails. A small proportion of probe stimuli prevented the crows to learn response patterns for those stimuli. Familiar training sample stimuli as well as probe sample stimuli were always followed by the same familiar test stimuli also used for training (see 'Training stimuli'). In either case, the crows were rewarded for every correct response to a match to promote category maintenance. Only responses to the first presentation of each unique probe stimulus were analyzed. During this first presentation of the probe stimulus, the crows were not able to learn a 'correct' response but had to infer category membership based on their previous knowledge acquired with training stimuli.

Data analysis

The percent correct responses, i.e. the number of correct trials divided by the total number of completed trials, was calculated as a measure of behavioral performance. Performance was calculated separately for up- and downward sweeping training sample stimuli and the classes of probe stimuli. To assess transfer of upward and downward FM categories, only the first trial for each unique probe FM stimulus was included. Probe trial performance therefore quantified the percentage of correctly answered first probe stimuli. This ensured that the crows could not learn how to respond to probe trials but relied on transferring their categorical perception.

Error types: The only type of error possible in the match condition is a type2-error (crow does not respond to match) because the trial ends after presentation of test1 (match). In the nonmatch condition, the crows only made type1-errors (false alarms; crow responds to nonmatch) because the crows always responded to either test1 or test2 (with the exception of a single trial across all sessions). The percent correct performance for match and nonmatch conditions separately therefore indicate all possible types of errors the crows made.
